# Overexpression of *Flii* during Murine Embryonic Development Increases Symmetrical Division of Epidermal Progenitor Cells

**DOI:** 10.3390/ijms22158235

**Published:** 2021-07-30

**Authors:** Gink N. Yang, Parinaz Ahangar, Xanthe L. Strudwick, Zlatko Kopecki, Allison J. Cowin

**Affiliations:** 1 Regenerative Medicine, Future Industries Institute, University of South Australia, Adelaide, SA 5000, Australia; gink.yang@mymail.unisa.edu.au (G.N.Y.); parinaz.ahangar@unisa.edu.au (P.A.); xanthe.strudwick@unisa.edu.au (X.L.S.); 2 Cell Therapy Manufacturing Cooperative Research Centre, Adelaide, SA 5000, Australia

**Keywords:** cell division, skin, progenitor cells, epidermis, embryonic

## Abstract

Epidermal progenitor cells divide symmetrically and asymmetrically to form stratified epidermis and hair follicles during late embryonic development. Flightless I (Flii), an actin remodelling protein, is implicated in Wnt/β-cat and integrin signalling pathways that govern cell division. This study investigated the effect of altering Flii on the divisional orientation of epidermal progenitor cells (EpSCs) in the basal layer during late murine embryonic development and early adolescence. The effect of altering Flii expression on asymmetric vs. symmetric division was assessed in vitro in adult human primary keratinocytes and in vivo at late embryonic development stages (E16, E17 and E19) as well as adolescence (P21 day-old) in mice with altered Flii expression (Flii knockdown: *Flii*^+/−^, wild type: WT, transgenic Flii overexpressing: *Flii^Tg^*^/*Tg*^) using Western blot and immunohistochemistry. *Flii*^+/−^ embryonic skin showed increased asymmetrical cell division of EpSCs with an increase in epidermal stratification and elevated talin, activated-Itgb1 and Par3 expression. *Flii^Tg^*^/*Tg*^ led to increased symmetrical cell division of EpSCs with increased cell proliferation rate, an elevated epidermal SOX9, Flap1 and β-cat expression, a thinner epidermis, but increased hair follicle number and depth. Flii promotes symmetric division of epidermal progenitor cells during murine embryonic development.

## 1. Introduction

Epidermal progenitor cells (EpSCs) divide both asymmetrically and symmetrically during embryonic skin development [1] These different forms of cell division maintain a pool of progenitor cells that can give rise to various compartments of the skin including the stratified epidermis and hair follicles [2]. Epidermal cellular division orientation is critical to the process of epidermal stratification, an essential morphogenic process required for the epidermis to act as a functional barrier to the external environment [3] and is implicated in cancer progression where normal asymmetric cell division (ACD) mechanisms are disrupted [4,5]. Prior to epidermal stratification, the majority of mitotic basal cell division occurs symmetrically with cells dividing parallel to the underlying basement membrane [6]. Over time, these symmetrically dividing multipotent stem cells develop to become short-lived EpSCs, generating daughters capable of asymmetric division while maintaining slow-cycling [7]. The formation of the epidermis begins at E9.5 and from E14 onwards, asymmetric basal cell division occurs with cells dividing perpendicular to the basement membrane and this is maintained until birth when the epidermis is fully mature [6]. Established through this stratification of the developing epidermis, the skin barrier is formed at E16.5 in Balb/c mice [8]. Beginning at E14.5, ACD is also adopted by developing EpSCs in the basal layer to form hair placodes while generating progenies retaining the adult stem cell features [7]. These placodes interact with the dermal papilla, which develops into hair pegs that continue to mature into hair follicles at E18.5 and become fully functional with the ability to produce hair fibre at P21 in rodents [9,10].

During late embryonic stages of development, when morphogenesis peaks, the majority of EpSCs go through ACD [2]. These EpSCs continue to develop into adult epidermal stem cells with diverse heterogeneity [11]. Nonetheless, foetal EpSCs all have a common proliferative character defined by the distinct expression of progenitor marker delta Np63 (∆Np63) and basal marker keratin 15 (K15) [12,13]. Previous studies have also implicated ∆Np63 as a key factor stimulating ACD during epidermal cell divisions [6,14]. Sex-Determining Region Y-Box 9 Protein (SOX9) has been shown to inhibit differentiation of EpSCs into keratinocytes by stimulating symmetric cell division (SCD) during stem cell self-renewal and cancer progression [15,16]. As a direct downstream target of beta-catenin (β-cat), upregulated SOX9 expression through constitutive β-cat activation has been found to enhance the colony-forming capacity of cancer cells from squamous cell carcinoma [17]. 

Identified as important regulators of mitotic spindle orientation, integrins participate in the establishment of cell adhesion and polarity in epithelia by interacting with the cytoskeleton, focal adhesion proteins and the extracellular matrix [18]. Extrinsic signals are thought to establish intrinsic polarity through integrin signal transduction during ACD. Together with partitioning defective proteins (PARs) [19], integrins are accepted to determine apical-basal polarity and cell fate during cytokinesis, and their expression is required to limit the proliferation and self-renewal of epidermal stem cells. The silencing of PARs results in an increase in the number of undifferentiated cells in the epidermis [20]. Mechano-sensing proteins including integrin-cytoskeleton regulators, focal adhesion (FA) proteins and actin-remodelling proteins also have an important role in stem cell division [21]. Following ligand-induced or force-dependent integrin activation, FA proteins are recruited to an activated integrin β1 (Itgb1) complex, at the cell cortex during mitosis, in order to direct signal transduction from activated Itgb1 to the mitotic spindle [18]. The transmembrane protein talin, links the F-actin polymers to the cytoplasmic Itgb1 hence resulting in the recruitment of other adaptor proteins including paxillin and vinculin, ultimately initiating focal adhesion kinase (FAK) and integrin-linked kinase (ILK) signalling [22].

The actin remodelling protein Flightless I (Flii) binds to actin structures and localizes to active cellularization regions during embryonic development [23]. Mutations in Flii homolog, *fli-1*, disrupt the anterior/posterior polarity, cytokinesis and ACD in germline development of *Caenorhabditis elegans* [24]. Additionally, the loss of *Flii* leads to embryonic lethality in both *Drosophila* and the mouse [25]. Flii expression increases during development and its over-expression has been shown to impair activation of epidermal stem cells, skin barrier development and regulate cellular processes by interacting with integrin-binding proteins including paxillin, vinculin and talin [26,27]. Moreover, the interplay of Flii with Flightless I associated protein 1 (Flap1 or LRRFIP1) has been found to regulate β-cat dependent transcription [28,29]. Flap1 is an important regulator of canonical Wnt-signalling (Wnt) and its interaction with β-cat results in an increased β-cat/lymphocyte enhancer-binding factor 1/T cell factor (LEF1/TCF) activated transcription via recruitment of the coactivator creb-binding protein (CBP)/p300 to the promoter in the nucleus [29,30]. Identified as a negative regulator of wound repair, reduced Flii levels improve wound contraction, epithelial proliferation and cell migration [31]. Overexpression of Flii during late gestation impairs skin barrier formation by decreasing the expression of tight-junction proteins in the epidermis [8]. Reduced Flii also increases laminin and Itgb1 protein levels in adult skin wounds via interacting with talin to activate integrins and promote integrin-mediated signalling pathways [26]. Flii has further been shown to regulate the regeneration of murine skin appendages with *Flii* overexpression leading to longer hair fibre lengths in regenerated hair follicles; increased claw regeneration with elevated epidermal β-cat expression and significantly thicker nails following proximal digit amputation [32,33].

As Flii has been shown to have a critical role in regulating embryonic development and EpSC activation the objective of this study was to investigate if altering Flii expression impacted EpSC division symmetry during late embryonic development and adolescence. The aim was to (1) determine the mitotic division patterns in adult murine primary keratinocytes in vitro; (2) assess the number of EpSCs, the proliferation rate and the symmetric pattern of division in EpSC of different embryonal stages in mice with altered Flii gene expression; (3) investigate the epidermal stratification and the morphogenesis between the stratified epidermis and hair follicles in these mice. Overall, we demonstrated that reduced levels of Flii promote asymmetrical cell division of EpSCs while Flii overexpression promotes symmetrical cell division with impacts on β-cat and SOX9 signalling.

## 2. Results

### 2.1. In Vitro Assessment of Cell Division and Associated Protein Expression in Adult Murine Primary Keratinocytes

**Figure 1:**
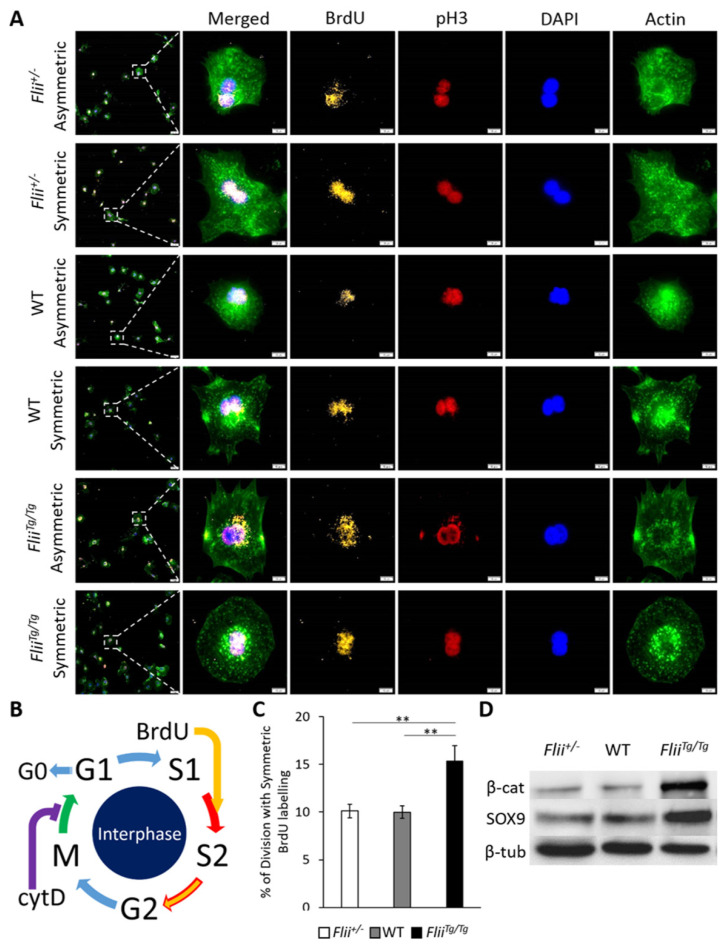
In vitro assessment of mitotic division in adult murine primary keratinocytes using BrdU-cytD pulse-chase labelling. (**A**) Representative images of proliferating keratinocytes going through asymmetric division (Asym) and symmetric division (Sym). Triple immunofluorescent staining of BrdU (pseudo-stained yellow) showing DNA synthesis in proliferating cell, pH3 (pseudo-stained red) identifying mitotic chromosomes during metaphase of proliferation and phalloidin (pseudo-stained green) identifying actin filaments. Nuclear DAPI staining shown as pseudo-stained blue. Asymmetrically dividing cells show skewed inheritance of BrdU-labelled DNA at the end of metaphase, symmetrically dividing cells shown equal inheritance of BrdU-labelled DNA at the end of metaphase. Arrest of cytokinesis by cytD is confirmed by phalloidin staining showing disrupted formation of actin filaments in granular shape. Scale bars, 5 μm. *Flii*^+/−^, Flii knockdown; WT, wild-type; *Flii^Tg^*^/*Tg*^, Flii overexpressing. (**B**) Schematic diagram summarizing the process of BrdU incorporation into DNA synthesis between S1 and S2 phase and the arrest of cytokinesis by cytD between M and G1 phase during mitotic division. (**C**) Measurement of mitotic cells going through symmetric division as % of division with symmetric BrdU-labelling. Data represent the mean from three independent experiments with an average of 30 mitotic events per group. Mean ± SEM. ** *p* < 0.005. (**D**) Western blot analysis of β-catenin, SOX9 and β-tubulin protein levels in primary mouse keratinocytes. Images represent one of three independent experiments.

To investigate if Flii plays a role in the division orientation of proliferative keratinocytes, the division pattern of primary keratinocytes isolated from *Flii*^+/−^, WT and *Flii^Tg^*^/*Tg*^ epidermis were characterized by BrdU-cytD assay. The pulse-chased cells were subsequently stained with anti-BrdU antibody to visualize the newly synthesized nucleus that inherited BrdU label asymmetrically or symmetrically. Importantly, arrested cytokinesis by cytD was confirmed by pH3 staining of duplicated yet unseparated chromatins as well as phalloidin staining of disrupted actin structure in granular form rather than fibrotic (Figure 1A,B). Asymmetric division resulting in only one nucleus inheriting BrdU label and symmetric division resulting in both nuclei inheriting BrdU label were observed in all three genotypes of cells (Figure 1A). No significant difference was observed in the percentage of ACD with BrdU labelling in between the three genotypes. However, a significantly increased percentage of SCD was found in *Flii^Tg^*^/*Tg*^ primary keratinocytes when compared to *Flii*^+/−^ and WT counterparts (Figure 1C). To further investigate the role of Flii in β-catenin regulated SOX9 expression at a protein level, the level of β-catenin and SOX9 in *Flii*^+/−^, WT and *Flii^Tg^*^/*Tg*^ primary mouse keratinocytes (n = 4) was characterized using Western blotting. Increased level of β-catenin protein was observed in *Flii^Tg^*^/*Tg*^ cells, which was accompanied by increased levels of SOX9 when compared to *Flii*^+/−^ and WT counterparts (Figure 1D and Figure S2).

### 2.2. Altering Flii Gene Expression Does Not Impact the Numbers of ΔNp63^+^K15^+^EpSCs

**Figure 2:**
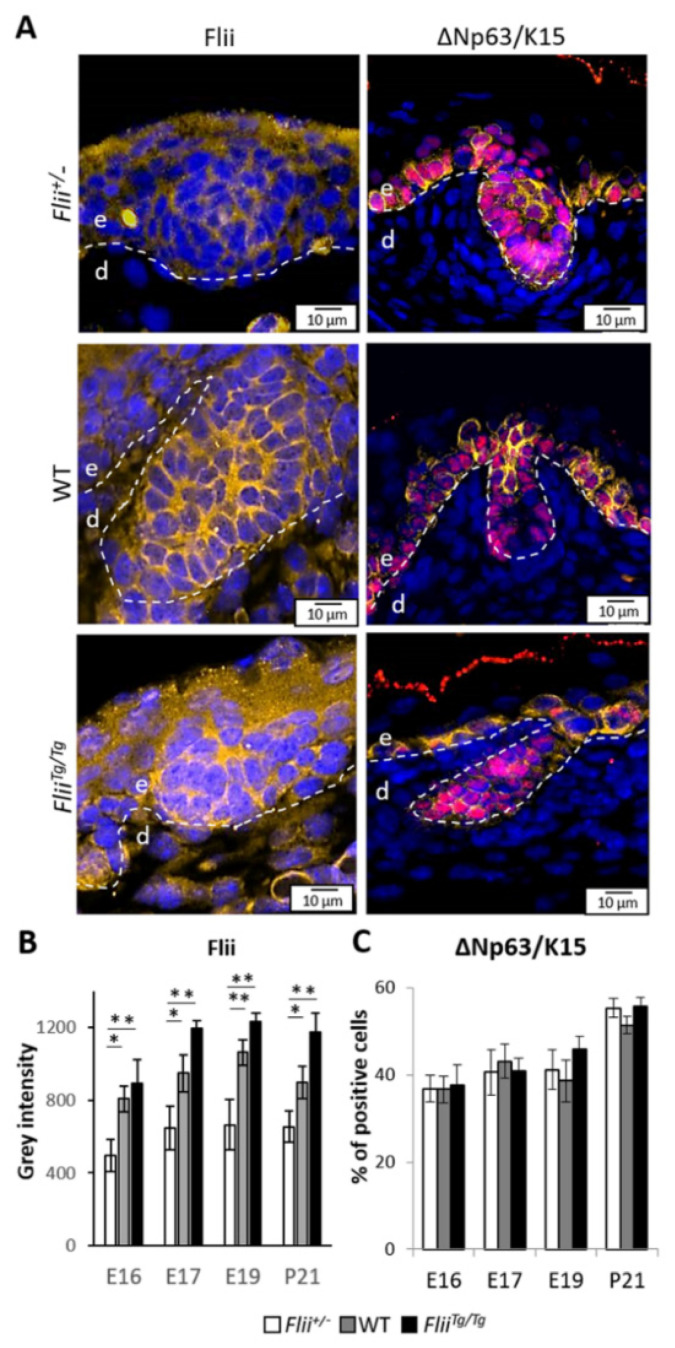
Alteration of Flii expression does not affect EpSC number during embryonic development. (**A**) Immunofluorescence detection of Flii, ∆Np63 and K15 positive cells in developing epidermis at E17. Flii shown as pseudo-stained yellow, ∆Np63^+^K15^+^ positive cells shown as pseudo-stained pink and yellow. Nuclear DAPI staining shown as pseudo-stained blue. The epidermal-dermal junction shown by white dash lines; e, epidermis; d, dermis. Scale bars, 10 μm. *Flii*^+/−^, Flii knockdown; WT, wild-type; *Flii^Tg^*^/*Tg*^, Flii overexpressing. Quantification of (**B**) Flii expression as grey intensity in the epidermis, (**C**) Number of progenitor cells as % of ∆Np63^+^K15^+^ cells in total basal cells at E16, E17, E19 and P21. N = 6. Mean ± SEM. * *p* < 0.05. ** *p* < 0.005.

EpSCs reside along with the basal layer of the developing epidermis and express ∆Np63 and K15 in both interfollicular and follicular regions [13]. *Flii* homozygous (*Flii^−^*^/−^) mice are embryonic lethal [25]. Therefore, to investigate if developmental preference in embryonic epidermis brought by differential *Flii* expression affects EpSC numbers, the proportion of ΔNp63^+^K15^+^ cells was first assessed within the entire population of epidermal cells in developing skin of *Flii*^+/−^, WT and *Flii^Tg^*^/*Tg*^ mice. The Flii protein levels have been extensively characterised in these mice in our previous studies to demonstrate a 0.5-fold reduction in *Flii*^+/−^ mice and a 1.5-fold increase in Flii expression in *Flii^Tg^*^/*Tg*^ mice [8,31,34]. Flii expression during development was confirmed to be significantly lower in *Flii*^+/−^ at E16 epidermis when compared to WT and *Flii^Tg^*^/*Tg*^ counterparts, while Flii over-expression was observed in *Flii^Tg^*^/*Tg*^ epidermis at E17 and E19 when compared to WT and *Flii*^+/−^ counterparts, peaking at E19. (Figure 2A,B). ΔNp63^+^K15^+^ EpSCs were found in the basal layer and HF of the developing epidermis in all strains. The percentage of EpSCs remained between 36.7–38.6% during late embryonic development (E16, E17, E19) in wild-type mice rising to 55.6% at P21. Altering the level of *Flii* expression did not significantly change the percentage of EpSCs within the epidermis (Figure 2C).

### 2.3. Overexpression of Flii Results in Proliferating Cells Preferably Undergoing Symmetrical Division While Reduced Flii Promotes Asymmetrical Division

**Figure 3:**
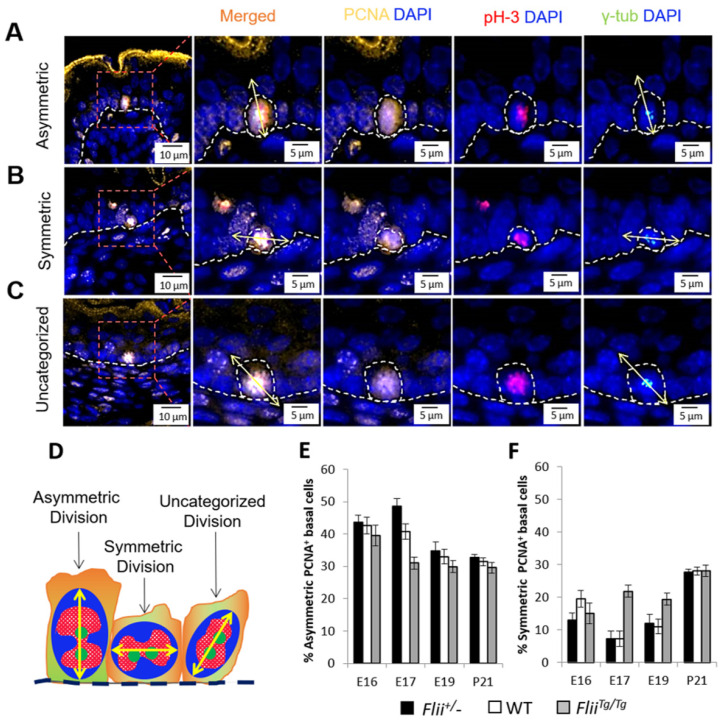
Altered expression of the *Flii* gene results in proliferating basal cells adopting different cell division patterns during late embryonic development. (**A**) Representative image of proliferating basal cell going through asymmetric division with division plane perpendicular to the basement membrane, (**B**) symmetric division with division plane parallel to the basement membrane and (**C**) uncategorized division with division plane diagonal to the basement membrane. Triple immunofluorescent staining of PCNA (pseudo-stained yellow) identifying proliferating cell, pH3 (pseudo-stained red) identifying mitotic chromosomes during metaphase of proliferation and γ-tub (pseudo-stained green) identifying centrosomal axis. Nuclear DAPI staining is shown as pseudo-stained blue. The epidermal-dermal junction is shown by white dash lines. Scale bars, 5 μm. (**D**) Cartoon summarizing the three division categories. (**E**) Quantification of proliferating basal cells going through the asymmetric division with perpendicular division plane and (**F**) symmetric division with parallel division plane and as % of total proliferating events with an average of 100 proliferating events per group at E16, E17, E19 and P21 in *Flii*^+/−^, Flii knockdown; WT, wild-type; *FIii^Tg/Tg^*, Flii overexpressing mouse skin. N = 6. Mean ± SEM.

**Figure 4:**
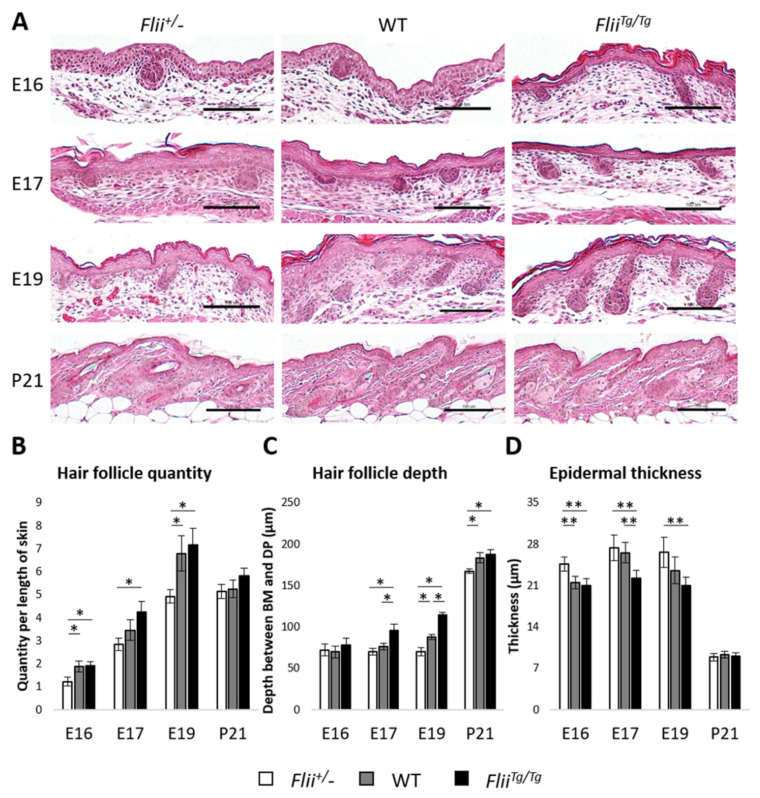
Morphogenesis of stratified epidermis and hair follicles is affected in mice expressing varying levels of Flii during late embryonic development. (**A**) Representative H&E images of the developing epidermis at different embryonic day including E16, E17, E19 and postnatal at P21. *Flii*^+/−^, Flii knockdown; WT, wild-type; *Flii^Tg^*^/*Tg*^, Flii overexpressing. Scale bars, 100 μm. (**B**) Hair follicle (including its infantile and juvenile forms as placodes and hair pegs) quantity was determined by measuring the number of developing hair follicle per length of skin (**C**) Hair follicle (including its infantile and juvenile forms as placodes and hair pegs) depth was determined by measuring the distance between basement membrane (BM) and dermal papilla (DP). (**D**) Epidermal thickness was determined by measuring the distance between BM and stratum granulosum in interfollicular regions across consistent length of epidermis at E16, E17, E19 and P21. N = 6. Mean ± SEM. * *p* < 0.05. ** *p* < 0.005.

To further visualise the difference in cell division pattern brought by differential Flii expression, the mitotic division plane of the proliferating cells was assessed in the developing epidermis within the proliferating basal layer where ΔNp63^+^K15^+^ EpSCs reside. PCNA^+^ cells in the skin sections were co-localized with phosphor-histone H3 (pH3) and γ-tubulin (γ-tub) to capture the dividing chromosomes and corresponding centrosomes, respectively (Figure 3A–C and Figure S1). The division orientation was determined from an average of 100 cells per group, by assessing the direction of the centrosome axis against the basement membrane as asymmetric (perpendicular to basement membrane), symmetric (parallel to basement membrane) or uncategorized division (Figure 3D) in both follicular and interfollicular regions (Figure 3A). In WT mice at E16 when the stratification process was commencing, 42.6% of cell divisions were classified as asymmetric while 19.6% of divisions were symmetric (Figure 3E). At E17, even fewer symmetric divisions were observed (7.3%) with the predominant form of cell division being asymmetric. However, by P21, when the epidermis was fully functional, the ratio of asymmetric to symmetric division was close to 1:1 (31.4% and 28.1%, respectively) (Figure 3F). Altering Flii gene expression had minimal effect on cell division orientation at E16 but significant changes were observed at E17 and E19. Overexpression of Flii (*Flii^Tg^*^/*Tg*^) led to a decrease in the numbers of cells undergoing asymmetric division at E17 (31.1% *Flii^Tg^*^/*Tg*^ vs. 40.8% WT) while a 3-fold increase in symmetric cell division was observed (21.8% *Flii^Tg^*^/*Tg*^ vs. 7.3% WT) (Figure 3E,F). This corresponded with a significantly thinner epidermis at E17 compared to WT (Figure 4A,D). When Flii levels were reduced (*Flii*^+/−^) an increase in asymmetric divisions were observed (48.6% *Flii*^+/−^ vs. 40.8% WT) at E17 (Figure 3E) while the number of symmetric divisions remained similar to those observed in WT mice (7.3%) (Figure 3F). This corresponded with a significantly thicker and more stratified epidermis at E17 compared to WT (Figure 4A,D). At E19, the percentage of symmetric divisions remained low (11.0%) in both WT and *Flii*^+/−^ epidermis, but higher numbers of symmetric divisions (19.4%) were again observed in *Flii^Tg^*^/*Tg*^ epidermis (Figure 3F). Consistently, thinner and less stratified epidermis was observed in *Flii^Tg^*^/*Tg*^ mice at E17 and E19 (Figure 4A,D). No significant differences were observed in asymmetric divisions between the three genotypes at E19 (Figure 3E). By P21, an adolescent stage when epidermal compartments are fully matured, the percentage of asymmetric and symmetric divisions were similar with both reaching to about 30%, with no significant differences observed between the three genotypes (Figure 3E,F).

### 2.4. Differential Flii Expression Directs Morphogenesis between Stratified Epidermis and Hair Follicles during Late Embryonic Progression

Using skin from the late embryonic and juvenile stages of Flii heterozygous (*Flii*^+/−^), wild-type (WT) and Flii overexpressing (*Flii^Tg^*^/*Tg*^) mice, we investigated the effect of *Flii* on the development of major epidermal components including the stratified epidermis and hair follicles. The number of emerging HFs, the depth of the HFs and epidermal thickness were analysed to assess the effect of Flii expression on the developmental preference of epidermal components. The formation of placodes was observed in the embryonic skin from all three genotypes of *Flii* mice at E16 (Figure 4A). The number of emerging placodes was significantly lower in *Flii*^+/−^ skin when compared to WT and *Flii^Tg^*^/*Tg*^ counterparts, while epidermal thickness was significantly decreased in *Flii^Tg^*^/*Tg*^ skin when compared to *Flii*^+/−^ and WT counterparts (Figure 4B,D). However, no significant difference in the depth of the emerging placodes was observed between the three genotypes at E16 (Figure 4C). These developmental preferences continued onwards at E17, where the number of hair pegs was significantly higher in *Flii^Tg^*^/*Tg*^ skin when compared to *Flii*^+/−^ counterpart, with the depth of hair peg increased and epidermal thickness lessened in *Flii^Tg^*^/*Tg*^ skin when compared to *Flii*^+/−^ and WT counterparts (Figure 4B–D). By E19, the number of hair pegs remained significantly lower in *Flii*^+/−^ skin when compared to WT and *Flii^Tg^*^/*Tg*^ counterparts. The depth of hair pegs was lowest in *Flii*^+/−^ skin, intermediate in WT and highest in *Flii^Tg^*^/*Tg*^ skin, respectively, while epidermal thickness remained lower in *Flii^Tg^*^/*Tg*^ skin when compared to *Flii*^+/−^ and WT counterparts (Figure 4B–D). By P21, the difference in the number of hair follicles and epidermal thickness was no longer significant, while the depth of hair follicles remained lower in *Flii*^+/−^ skin when compared to WT and *Flii^Tg^*^/*Tg*^ counterparts (Figure 4B–D).

### 2.5. Overexpression of Flii Results in Increased SOX9 Expression and Elevated Epidermal Cell Proliferation

**Figure 5:**
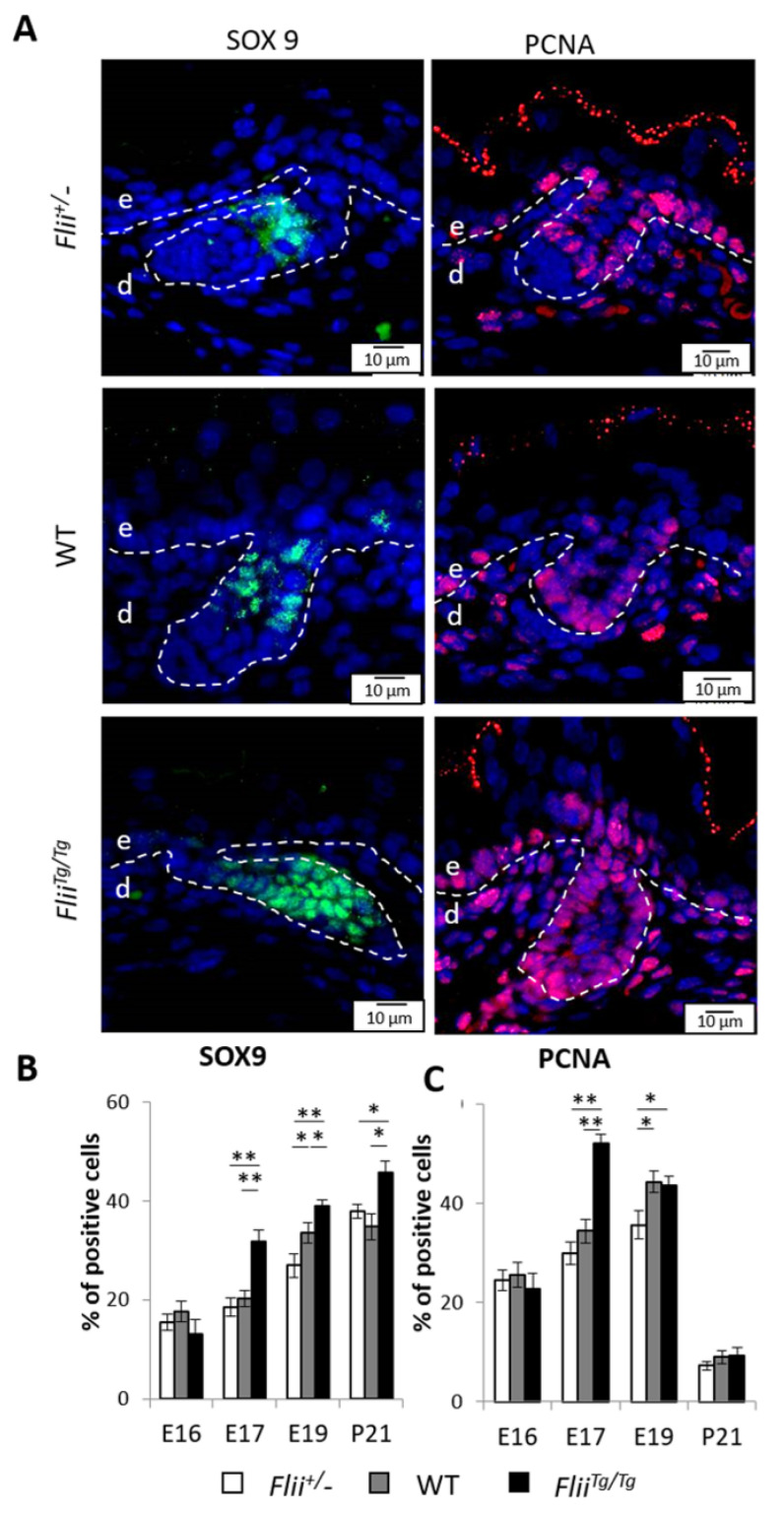
Overexpression of *Flii* gene results in increased number of SOX9^+^ cells and cell proliferation in basal epidermis during late embryonic development. (**A**) Immunofluorescence detection of SOX9 and PCNA positive cells in developing epidermis at E17. SOX9^+^ positive cells are shown as pseudo-stained green and PCNA^+^ positive cells are shown as pseudo-stained red. Nuclear DAPI staining shown as pseudo-stained blue. The epidermal-dermal junction is shown by white dash lines; e, epidermis; d, dermis. Scale bars, 10 μm. *Flii*^+/−^, Flii knockdown; WT, wild-type; *Flii^Tg^*^/*Tg*^, Flii overexpressing. Quantification of (**B**) SOX9 expression as % of SOX9^+^ cells and (**C**) PCNA expression quantified as % of PCNA^+^ cells in total basal cells at E16, E17, E19 and P21. N = 6. Mean ± SEM. * *p* < 0.05. ** *p* < 0.005.

Given that overexpression of *Flii* induced more HFs yet the proportion of progenitor cells remained unchanged, we next investigated if the cell division preferences in the proliferating basal layer and HFs, where ΔNp63^+^K15^+^ EpSCs reside, exhibited any differences. SOX9 has previously been shown to promote HF development, symmetrical cell division and self-renewal of stem cells during development [7]. To determine if altering *Flii* gene expression during epidermal development affects this downstream target of β-cat signalling, the number of SOX9^+^ cells were assessed in mice with differential expression of *Flii*. SOX9^+^ cells within the epidermis steadily increased during this developmental period (Figure 5A,B). While the numbers of SOX9^+^ cells were similar in all three genotypes at E16 (Figure 5A,B), the number of SOX9^+^ cells significantly increased in *Flii^Tg^*^/*Tg*^ skin from E17 onwards when compared to *Flii*^+/−^ and WT counterparts (Figure 5B). At E19, a significant difference in the number of SOX9^+^ cells was also observed between *Flii*^+/−^ and WT mice in the epidermis, with the least number of positive cells in *Flii*^+/−^ epidermis, intermediate in WT and highest in *Flii^Tg^*^/*Tg*^ epidermis (Figure 5B). By P21, the difference in SOX9^+^ cell numbers diminished between *Flii*^+/−^ and WT epidermis, while the highest number of SOX9^+^ cells remained in *Flii^Tg^*^/*Tg*^ epidermis (Figure 5B).

Previous studies have described a role for SOX9 in promoting cell proliferation, so we investigated if proliferating cell nuclear antigen expression (PCNA) expression was different between *Flii* mice genotypes during epidermal development (Figure 5A,C). Similar numbers of proliferating cells were detected between the three genotypes at E16, with the numbers steadily increasing from E16 to E19 (Figure 5C). At E17, a significant increase in the number of proliferating cells was observed in *Flii^Tg^*^/*Tg*^ epidermis when compared to *Flii*^+/−^ and WT counterparts, with most of the increase observed in the follicular epidermis (Figure 5C). The number of proliferating cells peaked in the *Flii*^+/−^ and WT epidermis at E19, with WT epidermis containing similar number of proliferating cells as *Flii^Tg^*^/*Tg*^ epidermis while *Flii*^+/−^ epidermis consistently showed the least (Figure 5C). By P21, the number of proliferating cells decreased in the epidermis in all three genotypes of mice with no significant difference observed between the groups (Figure 5C).

### 2.6. Overexpression of Flii Results in Increased Epidermal Flap1 and β-Cat Expression during Late Embryonic Development

**Figure 6:**
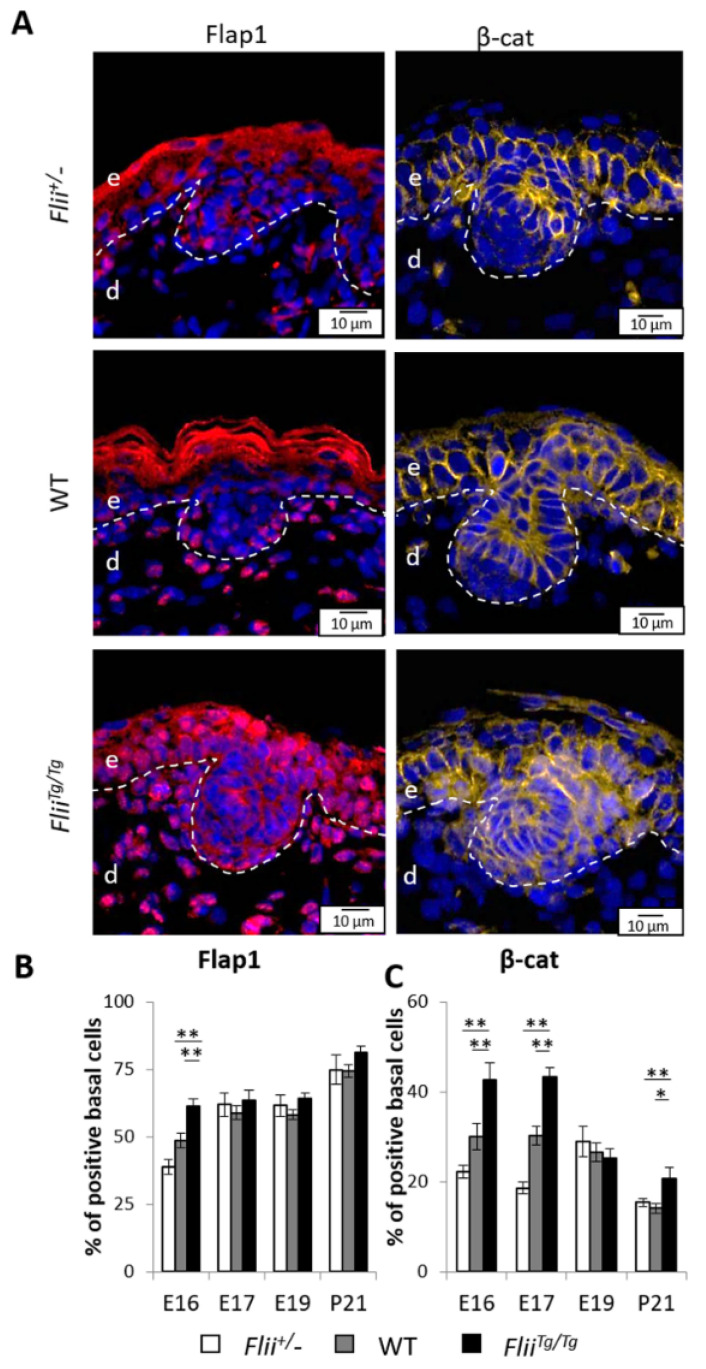
Expression of Flap1 and β-cat in the epidermis of mice expressing varying levels of *Flii* during late embryonic development. (**A**) Immunofluorescence of Flap1 and β-cat in developing epidermis at E16. Flap1 is shown as pseudo-stained red, and β-cat is shown as pseudo-stained yellow. Nuclear DAPI staining is shown as pseudo-stained blue. The epidermal-dermal junction is shown by white dash lines; e, epidermis; d, dermis. Scale bars, 10 μm. *Flii*^+/−^, Flii knockdown; WT, wild-type; *Flii^Tg^*^/*Tg*^, Flii overexpressing. Quantification of (**B**) Flap1 expression as % of positive cells with nuclear FLAP expression and (**C**) β-cat expression as % of positive cells with nuclear β-cat expression in total basal cells at E16, E17, E19 and P21. N = 6. Mean ± SEM. * *p* < 0.05. ** *p* < 0.005.

Flap1, an antagonist to Flii, is an important enhancer of β-cat stabilization and β-cat/LEF1/TCF activated transcription [30]. To determine whether *Flii* affected Flap1 and β-cat stabilization in the developing epidermis, the expression of these two proteins were measured as the number of positive basal cells with Flap1 or β-cat, respectively, in skin of *Flii*^+/−^, WT and *Flii^Tg^*^/*Tg*^ mice. Consistent with previous finding where Flap1 was detected in keratinocytes and fibroblasts [35], embryonic Flap1 expression was detected throughout the whole skin with apparent nuclear and cytoplasmic expression in the epidermis including the interfollicular and follicular regions (Figure 6A,B). At E16 the cytoplasmic expression of Flap1 was weak while the number of positive cells with basal nuclear Flap1 expression was found to be the highest in *Flii^Tg^*^/*Tg*^ epidermis, intermediate in WT and lowest in *Flii*^+/−^ epidermis (Figure 6A–C). From E17 to E19, Flap1 expression was mainly detected in nuclear form, with no apparent difference in the number of positive cells from all three genotypes. By P21, the number of positive cells with basal nuclear Flap1 expression reached the highest level at approximately 75% of positive basal cells in all three genotypes (Figure 6A,B). β-cat staining revealed predominant expression at cell membrane junctions throughout the epidermis and nuclear expression in basal epidermal cells from E16 (Figure 6A,C). The number of β-cat positive cells was the lowest in *Flii*^+/−^ epidermis, intermediate in WT and highest in *Flii^Tg^*^/*Tg*^ epidermis at both E16 and E17, with similar numbers between each embryonic day in different genotypes (Figure 6A,C). β-cat expression was relatively stable throughout the epidermal development on both WT and *Flii*^+/−^ mice until an observed decrease at P21, however *Flii^Tg^*^/*Tg*^ mice epidermis displayed a significant drop in β-cat expression during development and still had significantly increased number of β-cat positive basal cells at P21 compared to WT and *Flii*^+/−^ counterparts (Figure 6A,C).

### 2.7. Reduced Flii Levels Lead to Increased Epidermal Cell Differentiation during Late Embryonic Development

**Figure 7:**
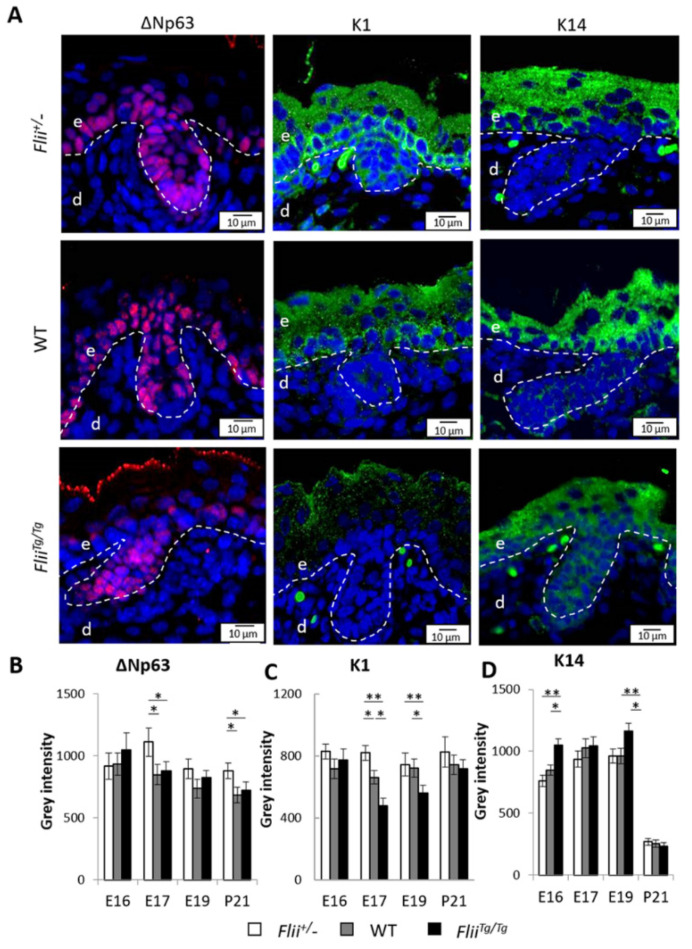
Flii deficiency results in increased differentiation potential in the epidermis during late embryonic development. (**A**) Immunofluorescence of ∆Np63 and K1 in developing epidermis at E17 and K14 at E19. ∆Np63 is shown as pseudo-stained red, K1 and K14 are shown as pseudo-stained green. Nuclear DAPI staining is shown as pseudo-stained blue. The epidermal-dermal junction is shown by white dash lines; e, epidermis; d, dermis. Scale bars, 10 μm. *Flii*^+/−^, Flii knockdown; WT, wild-type; *Flii^Tg^*^/*Tg*^, Flii overexpressing. Quantification of (**B**) ∆Np63 expression (**C**) K1 expression and (**D**) K14 expression as grey intensity in the epidermis at E16, E17, E19 and P21. N = 6. Mean ± SEM. * *p* < 0.05. ** *p* < 0.005.

Independent ∆Np63 expression is an indicator of a proliferative potential of dividing epidermal cells during asymmetric division [6,36]. ∆Np63 expression was significantly elevated in *Flii*^+/−^ epidermis exhibiting high numbers of asymmetric division at E17 (Figure 7A,B). While no significant difference in ∆Np63 expression was found at E16 or E19 between the genotypes, ∆Np63 expression was significantly higher in *Flii*^+/−^ epidermis when compared to WT and *Flii^Tg^*^/*Tg*^ counterparts at P21 (Figure 7B). Expression of keratin 1 (K1) and keratin 14 (K14) differentiation markers distinguish mature basal and suprabasal epidermis, respectively. These markers were examined in the epidermis of *Flii*^+/−^, WT and *Flii^Tg^*^/*Tg*^ mice (Figure 7A,C,D). *Flii* overexpression led to decreased suprabasal K1 expression at E17 and E19 (Figure 7A,C). K1 expression was constantly higher than that observed in WT and *Flii^Tg^*^/*Tg*^ mice (Figure 7C). Consistent with previous study where K14 expression was found to be ubiquitous in embryonic epidermis, indicating progenitor-like potential of developing epidermal cells [37], epidermal K14 expression was elevated in in *Flii^Tg^*^/*Tg*^ during E16 and E19 (Figure 7D). By P21, K1 expression was maintained at same level as during epidermal development while K14 expression was significantly reduced in all genotypes (Figure 7C,D).

### 2.8. Reduced Flii Results in Increased Talin, Activated-Itgb1 and Par3 Expression in the Epidermis during Late Embryonic Development

**Figure 8:**
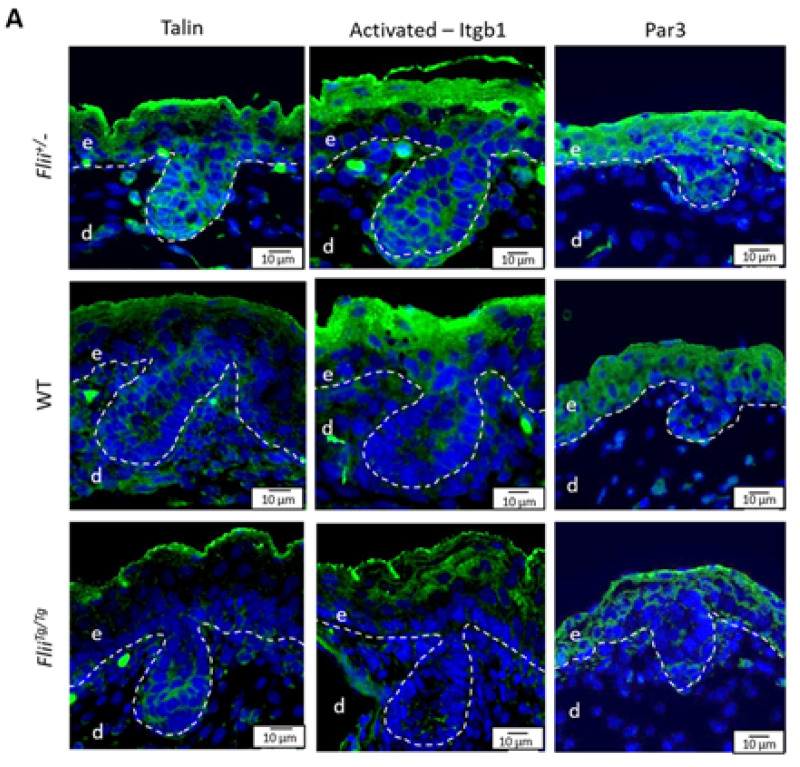

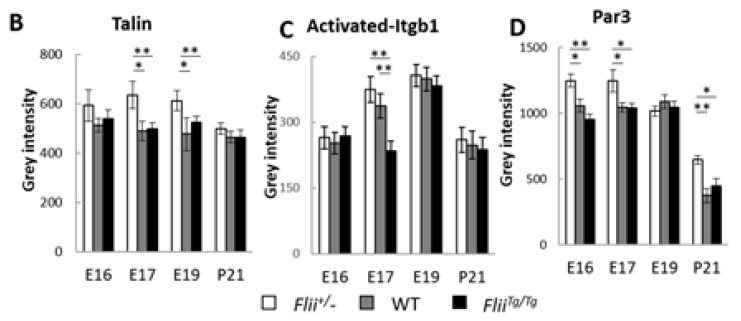
Expression of talin, activated-Itgb1 and Par3 in the epidermis of mice expressing varying levels of *Flii* during late embryonic development. (**A**) Immunofluorescence of talin and activated-Itgb1 in developing epidermis at E17 and Par3 at E16. Talin, Itgb1 and Par3 are shown as pseudo-stained green. Nuclear DAPI staining is shown as pseudo-stained blue. The epidermal-dermal junction is shown by white dash lines; e, epidermis; d, dermis. Scale bars, 10 μm. *Flii*^+/−^, Flii knockdown; WT, wild-type; *Flii^Tg^*^/*Tg*^, Flii overexpressing. Quantification of (**B**) Talin expression (**C**) Itgb1 expression and (**D**) Par3 expression as grey intensity in the epidermis at E16, E17, E19 and P21. N = 6. Mean ± SEM. * *p* < 0.05. ** *p* < 0.005.

Integrin-dependent cell adhesion at the basement membrane plays a positive role in the differentiation of epithelial cells. Acting in concert with cell polarity regulator Par complex, integrins have been shown to induce ACD and alter the outcome of cell lineage in adult epithelial stem cells [20]. Talin, an important integrin-binding protein and Itgb1 activator, was previously identified as a Flii binding partner [26]. To determine whether *Flii* affects the integrin-dependent mechanism required for asymmetric division in the developing epidermis, the expression of talin, activated-Itgb1 and Par3 were measured by the fluorescent intensity in skin of *Flii*^+/−^, WT and *Flii^Tg^*^/*Tg*^ mice during epidermal development (Figure 8A–D). Talin and Itgb1 expression were detected throughout the whole skin with clear expression within the epidermis including the interfollicular and follicular regions (Figure 8A). Epidermal talin and activated-Itgb1 expression were maintained at a similar level from E16 to P21 in all three genotypes of mice. While no significant difference in talin and activated-Itgb1 expression was observed at E16 in between the three genotypes (Figure 8B,C), *Flii*^+/−^ epidermis had significantly higher talin and activated-Itgb1 expression at E17 when compared to WT and *Flii^Tg^*^/*Tg*^ counterparts, with activated-Itgb1 expression being the lowest in *Flii^Tg^*^/*Tg*^ epidermis (Figure 8A–C) which was in line with previous findings in adult wounded skin [26]. By E19, talin expression remained the highest in *Flii*^+/−^ epidermis when compared to WT and *Flii^Tg^*^/*Tg*^ counterparts, while the differences in epidermal Itgb1 expression between the three genotypes could no longer be seen. By P21, no significant difference in either talin or Itgb1 expression was found between the three genotypes. The expression of Par3 was found to be mainly in the epidermis with much higher expression during development than adolescent skin. Unlike talin and Itgb1, the dose-dependent effect of *Flii* gene expression on Par3 expression and cell polarity started earlier than E17. At E16, *Flii*^+/−^ epidermis showed significantly increased Par3 expression when compared to WT and *Flii^Tg^*^/*Tg*^ counterparts, and same results were found at E17 (Figure 8A,D). By E19 no significant difference in Par3 expression was observed in between the three genotypes. At P21, elevated level of Par3 expression was re-established in *Flii*^+/−^ epidermis when compared to WT and *Flii^Tg^*^/*Tg*^ counterparts.

## 3. Discussion

Previous studies have shown that the level of *Flii* expression can affect both wound repair and tissue regeneration, both processes which are heavily reliant on the division and differentiation of epidermal progenitor/stem cells from their inactive niches [31,33,38]. Wound repair requires EpSCs differentiation for re-establishment of the barrier function, a process contributed mainly via the ACD of the progenitor cells [39]. Tissue regeneration, however, requires EpSCs to temporarily proliferate without losing their progenitor properties, a process that depends on the SCD of the progenitor cells [39]. The molecular basis for balancing the different division outcomes is yet to be understood completely.

Consistent with our previous finding showing delayed development of the epidermal skin barrier function in *Flii^Tg^*^/*Tg*^ embryonic skin at E17 [8] while reducing Flii decreased HF regeneration in adult rodents [32], the morphological results presented in this study suggest that the majority of *Flii^Tg^*^/*Tg*^ basal EpSCs follow symmetrical to develop down HF formation rather than asymmetrical cell division required for epidermal stratification. Our results indicate that the percentage of ∆Np63^+^K15^+^ progenitor cells do not differ in the embryonic epidermis of the three *Flii* genotypes, suggesting that *Flii* expression does not alter the developmental rate of EpSCs. As a downstream effector of β-cat dependent transcription, SOX9 expression is a positive modulator of SCD [7]. Overexpression of *Flii* led to increased SCD of proliferating basal cells shown by elevated SOX9 and PCNA expression during epidermal development and concurrent increased expression of K14 compared to wild-type counterparts during late embryonic development. Interestingly, *Flii* deficiency resulted in significantly increased ACD and increased K1 expression compared to wild-type and *Flii* over-expressing counterparts highlighting the important role that differential Flii levels play during epidermal development.

To better understand the effects of Flii on EpSCs cell division we examined the effects of differential levels of Flii on its signalling partner, Flap-1 expression, during epidermal development. Previous studies have shown that Flap1 directly interacts with Flii [29,30] while administration of recombinant-Flap1 to wounds in vivo directly reduced Flii levels [35]. As a naturally occurring antagonist to Flii, it is possible that Flap1 is expressed to neutralize the effects brought by *Flii* expression at a specific embryonic stage of epidermal development. Consequently, significantly lower nuclear expression of Flap1 in the basal epidermis was found in *Flii*^+/−^ embryonic skin at E16 when compared to WT mice skin while expression of Flap1 was significantly higher in the skin of *Flii^Tg^*^/*Tg*^ mice compared to both *Flii*^+/−^ and WT mice. Flap1 has been shown to interact with β-cat and lead to increased β-cat-dependent transcription activation in the nucleus, a process that promotes increased canonical Wnt pathway signalling [28]. Indeed, spatial and temporal regulation of Wnt/β-cat and β-cat-independent Wnt signalling during skin development has been shown to regulate cell division, polarity and tissue homeostasis [40].

Our results suggest that the antagonizing effects of Flap1 may have outweighed the negative effect brought by Flii during Wnt/β-cat signalling, resulting in elevated β-cat stabilization. Consequently, low, intermediate and high levels of nuclear β-cat were found in *Flii*^+/−^, WT and *Flii^Tg^*^/*Tg*^ skin, respectively. Although Flap1 levels remain similar in all three genotypes from E17 onwards, β-cat levels differ, respectively to Flap1 till E19 suggesting that β-cat stabilization takes place earlier during development and subsides by E19. Our finding of increased epidermal β-cat expression in *Flii^Tg^*^/*Tg*^ skin and keratinocytes suggests an important role for Flii regulation in Wnt/β-cat signalling pathway. Given that increased SOX9, an ER-regulated gene [41,42], was found in *Flii^Tg^*^/*Tg*^ epidermis when compared to *Flii*^+/−^ and WT counterparts, while Flii is an estrogen receptor coactivator, it is possible that elevated nuclear Flii promotes increased SOX9 at the transcriptional level which may also explain the increased number of PCNA^+^ cells observed in *Flii^Tg^*^/*Tg*^ embryonic mice skin secondary to increased SOX9 signalling that drives increased SCD. These findings are opposite to the observations in the colon epithelial cells where *Flii* over-expression was found to inhibit Wnt/β-cat signalling [43], suggesting cell and tissue-specific responses in Flii regulation of Wnt signalling and cell division during epidermal/epithelial development. 

Apart from influencing division pattern through Flap1 and β-cat signalling in the nucleus, Flii may also exert its modulatory effect via integrin-linked mechanisms, as ACD is highly dependent on cell polarity and relationship with the microenvironment [44]. Increased talin and Itgb-1 expression were observed in *Flii*^+/−^ mice epidermis, which together with increased Par3 levels and ACD suggest that the dividing cells in *Flii*^+/−^ mice epidermis adopted the apical-basal polarity. Concurrent with significantly higher ∆Np63 and K1 expression observed in *Flii*^+/−^ epidermis, these findings suggest that differentiation potential of EpSCs is elevated when Flii is reduced during morphogenesis. Indeed, ameliorated levels of talin in *Flii*^+/−^ epidermis may lead to the activation of Itgb1 which subsequently signals downstream effectors to enhance ACD of the proliferating EpSCs in the developing epidermis. Consistent with our previous findings demonstrating slower development of intact skin barrier during development and post injury accompanied with slower healing in *Flii^Tg^*^/*Tg*^ mice, results of this study are in agreement showing increased epidermal SCD in *Flii* over-expressing animals. Future studies should explore the distribution of epidermal growth factor receptor in *Flii^Tg/Tg^* mice skin and investigate if increased SCD pattern in *Flii^Tg^*^/*Tg*^ mice is an adaptation to delay the onset of cancers as increased Flii levels have been suggested to promote skin, colon and breast cancer development [45,46,47]. 

## 4. Conclusions

Our findings provide insights into the modulatory effect of Flii on EpSCs during late embryonic development although the exact mechanism underpinning Flii effects on cell division pattern is yet to be identified. While reduced Flii levels promote the microenvironment supportive of increased cell polarity and subsequent ACD, high Flii levels result in increased SCD mediated via effects on Wnt/β-cat signalling pathway. These alternative division pathways may influence EpSC differentiation and suggest that Flii may be a key regulator of epidermal cell division in specific cellular environments.

## 5. Materials and Methods

### 5.1. Animal Studies

All experiments and maintenance of mice were conducted according to Australian Standards for Animal Care under protocols approved by the Child Youth and Women’s Health Service Animal Ethics Committee (WCHN) and carried out in accordance with the Australian code of practice for the care and use of animals for scientific purpose (AE1019/10/18). All mouse strains were congenic on the BALB/c background. *Flii* deficient mice were generated by switching a null allele (*Flii*^tm1Hdc^) with an endogenous allele (*Flii^+^*) locus, with animals heterozygous for this mutation designated *Flii*^+/−^ [25]. The animals with one WT copy of the *Flii* gene and one mutant copy of the *Flii* gene express no more than 50% of the normal *Flii* gene expression [25]. WT (wild-type) littermates to *Flii*^+/−^ mice used as WT control animals. Transgenic *Flii* overexpressing mice (strain name: (Tg1FLII) 2Hdc) were generated by incorporating a 17.8-Kb fragment of a human cosmid clone that spans the entire *FLII* locus, with animals homozygous for the transgene in addition to the endogenous *Flii* allele designated *Flii^Tg^*^/*Tg*^. Details regarding to the generation of the transgenic mice strains were described previously, showing elevated levels of Flii protein in various tissues including skin [34]. An upregulation of Flii protein levels was confirmed using semi-quantitative Western analysis that showed total (mouse + human) protein levels up to 1.52-fold greater than wild-type levels [34]. Fetal skin collected at E16, E17, E19 of gestation and from P21 pups (n = 6). All skin was fixed in 10% formalin overnight, followed by dehydration through ethanol and xylene, and embedded in paraffin. 

### 5.2. Primary Murine Keratinocyte Isolation and Culturing

Dorsal skin was harvested from euthanized from *Flii*^+/−^, WT and *Flii^Tg^*^/*Tg*^ mice skin (age 8–10 weeks) and sterilized in iodine 10% antiseptic saline solution. Skin was cut into 1 cm wide squares and digested in serum-free DMEM (Sigma, Melbourne, Australia) buffered Dispase II (0.05 g/mL, Roche Diagnostics, Sydney, Australia) solution overnight at 4 °C, followed by removal of dermis the next day. The epidermal tissue was then digested in 0.25% trypsin/HBSS (Sigma) for 20 min at 37 °C, followed by addition of FBS (1:10) and filtering through a 70 μm and 40 μm nylon strainer (BD Biosciences, North Ryde, Australia). Isolated cells were plated into T75 flasks pre-coated with fibronectin and collagen (50 μg/mL, Invitrogen, Mount Waverley, Australia) containing low calcium Epilife medium (10% FBS in Epilife, Thermofisher, New South Wales, Australia) and cultured for 2 weeks until next passage. 

### 5.3. BrdU-Cytochalasin D Pulse-Chase Labelling of Murine Primary Keratinocytes

Primary keratinocytes from *Flii*^+/−^, WT and *Flii^Tg^*^/*Tg*^ mice skin were plated on glass coverslip in the well of a 6-well plate at a density of 20,000 cells in Epilife medium containing 10 μM BrdU (Sigma-Aldrich, Melbourne, Australia) as previously described [48]. Glass coverslips were pre-coated with fibronectin and collagen (50 μg/mL, Invitrogen, Australia). BrdU was allowed to incorporate into the DNA synthesis (pulse-process) for a period of 24 hr followed by washing in sterile PBS twice. New Epilife medium containing 5 μM cytochalasin D (cytD) (Sigma-Aldrich, Melbourne, Australia) was added to the cells and allowed to arrest cytokinesis (chase-process) for a period of 24 hr followed by washing in sterile PBS twice. 

### 5.4. Immunohistochemistry

Paraffin-embedded skin samples were sectioned (4 μm) prior to antigen retrieval using citrate buffer and trypsin as described previously [32]. Following antigen retrieval, 3% normal goat serum diluted in phosphate-buffered saline was used for blocking for 30 min. Primary antibodies including Flii (1:200, Santa Cruz Biotechnology, Dallas, TX, USA), Flap/LRRFIP1 (1:400, Bioss, Woburn, MA, USA), PCNA (1:200, Santa Cruz Biotechnology), K15 (1:400, Abcam, Cambridge, UK), ∆NP63 (1:400, Abcam, Cambridge, UK), K1 (1:200, Abcam, Cambridge, UK), K14 (1:100, Abcam, Cambridge, UK), β-catenin (1:200, Santa Cruz Biotechnology, Dallas, USA), SOX9 (1:1000, Abcam, Cambridge, UK), BrdU (1:500, Sigma Aldrich, Melbourne, Australia), pH3(s28) (1:400, Abcam, Cambridge, UK), γ-tubulin (1:500, Abcam, UK), PAR3 (1:500, Millipore, Sydney, Australia), Talin (1:500, Millipore, Sydney, Australia), and activated–Itgb1 (1:400, Merck, Kenilworth, NJ, USA) were diluted in the blocking buffer and applied. Species-specific Alexa Fluor 488, 568 or 633-conjugated secondary antibodies (1:400, Invitrogen, Sydney, Australia) were diluted in phosphate-buffered saline and applied for detection. For detection of actin, Oregon Green 488 Phalloidin (1:400, Thermofisher, Sydney, Australia) directly conjugated antibody was used in combination with secondary antibody. Nuclear counterstain 4,6-diamidino-2-phenyindole (DAPI) was applied last. Stained samples were imaged, followed by measurement of grey intensity using Olympus CellSens Dimension software.

For immunocytochemistry following BrdU-cytD pulse-chase assay, the cells were washed twice in washing buffered (0.5% BSA in PBS) and fixed in 4% paraformaldehyde. Fixed cells were permeabilized in buffer (0.2% Triton X-100 in washing buffer) followed by blocking in washing buffer. Primary antibodies including BrdU (1:400, Sigma-Aldrich) and pH3(s28) (1:400, Abcam, Cambridge, UK) were diluted in washing buffer and applied. Species-specific Alexa Fluor 568 or 633-conjugated secondary antibodies (1:400, Invitrogen, Australia) were diluted in washing buffer and applied along with Oregon Green 488 Phalloidin (1:400, Thermofisher, Australia) and DAPI. Stained cells were imaged, followed by counting of positive cells using Olympus CellSens Dimension software.

### 5.5. Protein Isolation and Western Blot

Protein was extracted from *Flii*^+/−^, WT and *Flii^Tg^*^/*Tg*^ adult murine primary keratinocytes as described previously [26]. Following brief homogenization in lysis buffer (50 mM Tris pH 7.5, 1 mM EDTA, 50 mM NaCl, 0.5% Triton X-100) containing protease inhibitor tablet (1 per 10 mL; Complete Mini (Roche, Indianapolis, IN, USA). Samples were centrifuged and supernatants collected. In total, 5 mg of protein was run on 10% SDS-PAGE gels at 100V for 1 h. and transferred to nitrocellulose membrane using standard Towbins Buffer with 20% Methanol at 100V for 1 h. Following blocking in 15% milk-blocking buffer for 10 min. Primary antibodies including Flii (1:200, Santa Cruz Biotechnology, Dallas, USA), β-catenin (1:200, Santa Cruz Biotechnology, Dallas, USA) and SOX9 (1:300, Abcam, Cambridge, UK) were diluted in buffer and applied to the membrane at 4 °C overnight. Species-specific secondary horseradish peroxidase-conjugated antibodies were diluted in 5% milk-blocking buffer and applied to the membrane at room temperature for 1 h. Protein bands were detected using Super Signal West Femto (Pierce Biotechnology, Rockford, IL, USA) and visualized with GeneSys analysis software (Syngene, Frederick, MD, USA).

### 5.6. Data Collection and Statistical Analysis

Grey intensity measurement was measured in epidermal regions including interfollicular epidermis and HFs, with the background intensity deducted from statistical analysis. Positive cells were counted from the basal layer of the epidermis and the HF compartments, the percent of positive cells was then calculated as the number of positive cells over the total number of cells in those regions. In vivo assessment of division direction was from an average of 100 proliferating cells in 6 individual mice per genotype at any embryonic stage. BrdU pulse-chase assay was assessed on an average of 40 proliferating cells from 9 different random regions on the slide of per experimental group. Data was analysed using the Student’s *t*-test to compare between two groups. A *p*-value of <0.05 was considered significant.

## Data Availability

The data that support the findings of this study are available from the corresponding author, AJC, upon reasonable request.

## Supplementary Materials

The following are available online at https://www.mdpi.com/article/10.3390/ijms22158235/s1.
