# Supplementary material for: Overexpression of Flii during Murine Embryonic Development Increases Symmetrical Division of Epidermal Progenitor Cells
Source: Int J Mol Sci. 2021 Jul 30;22(15):8235. doi: 10.3390/ijms22158235 (PMC8348627; doi:10.3390/ijms22158235)
Supplement: Supplementary file 1 [file ijms-22-08235-s001.zip › ijms-1288538-supplementary.pdf]

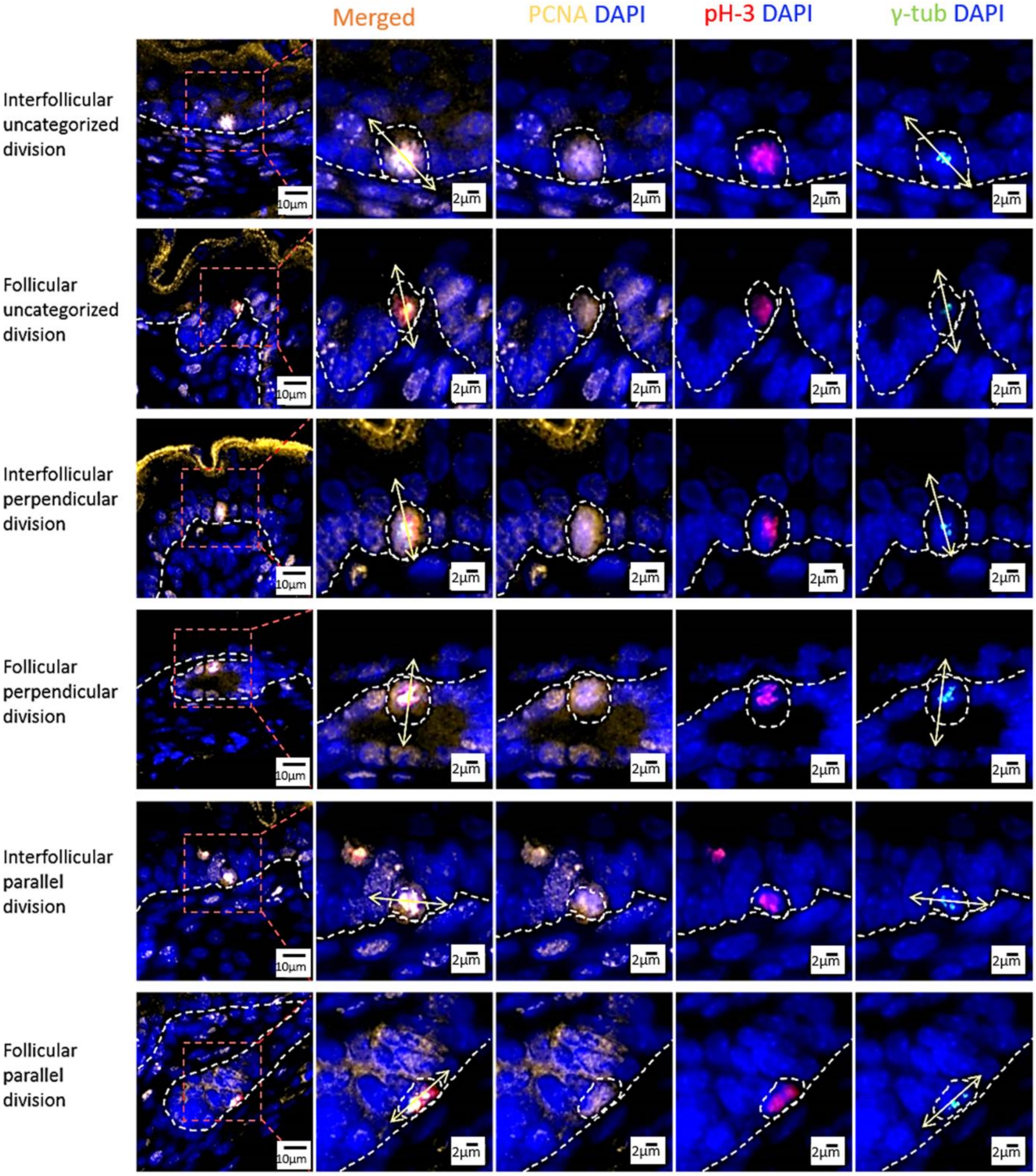

Figure S1. Altered expression of *Flii* gene results in proliferating basal cells adopting different cell division patterns in both interfollicular and follicular regions. Representative images of proliferating basal cell going through uncategorized division with division plane diagonal to the basement membrane, asymmetric division with division plane perpendicular to the basement membrane and symmetric division with division plane parallel to the basement membrane and. Triple immunofluorescent staining of PCNA (pseudo-stained yellow) identifying proliferating cell, pH3 (pseudo-stained red) identifying mitotic chromosomes during metaphase of proliferation and γ-tub (pseudo-stained green) identifying centrosomal axis. Nuclear DAPI staining shown as pseudo-stained blue. The epidermal-dermal junction shown by white dash lines; e, epidermis; d, dermis. Scale bars, 2μm.

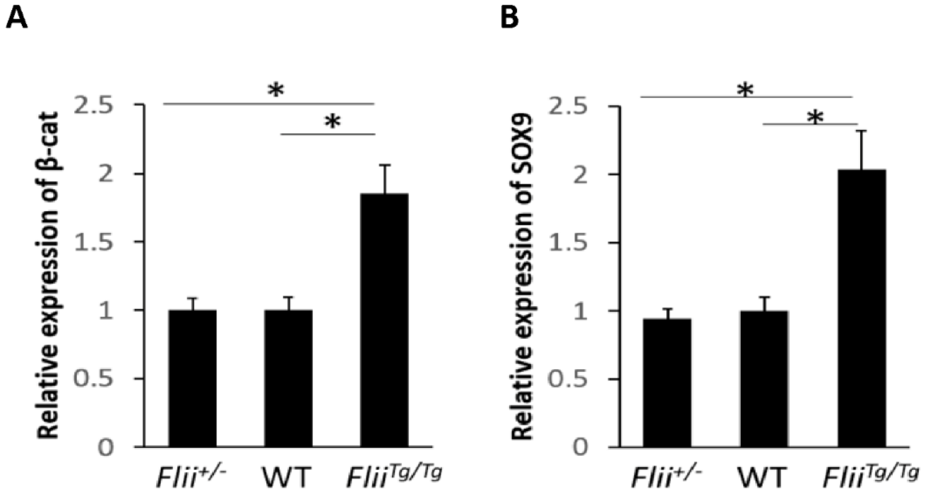

Figure S2. Comparison of protein levels via Western Blot on lysates of primary keratinocytes (P2) isolated from skins of Flii+/-, WT and FliiTg/Tg mice at 8 weeks of age. Quantification of relative protein levels as fold change of (A) β-catenin and (B) SOX9 to β-tubulin protein from each genotype normalized to mean WT value. Mean +/-SEM. *P<0.05.
